# New insights about functional and cross-reactive properties of antibodies generated against recombinant TbpBs of *Haemophilus parasuis*

**DOI:** 10.1038/s41598-017-10627-0

**Published:** 2017-09-04

**Authors:** Bibiana Martins Barasuol, João Antônio Guizzo, Jamie Elisabeth Fegan, Sonia Martínez-Martínez, Elías Fernando Rodríguez-Ferri, César Bernardo Gutiérrez-Martín, Luiz Carlos Kreutz, Anthony Bernard Schryvers, Rafael Frandoloso

**Affiliations:** 1 Laboratory of Microbiology and Advanced Immunology, Faculty of Agronomy and Veterinary Medicine, University of Passo Fundo, Passo Fundo, 99052-900 Brazil; 2 Department of Microbiology & Infectious Diseases, Faculty of Medicine, University of Calgary, Calgary, T2N 4N1 Alberta Canada; 3 Unidad de Microbiología e Inmunología, Departamento de Sanidad Animal, Facultad de Veterinaria, Universidad de León, 24007 León, Spain

## Abstract

Vaccines have become fundamental in the control and elimination of Glässer Disease, a systemic disease of pigs caused by *Haemophilus parasuis*. The classic vaccines available for prevention of this infection were developed without a robust knowledge about host immunological mechanisms. In this study, we demonstrated the presence of cross-reactive epitopes on both the N-lobe and C-lobe of variants of transferrin binding protein B (TbpBs) expressed on the surface of 6 virulent serovars of *H. parasuis*. Antibodies against TbpB-derived antigens were capable of increasing the phagocytic capacity of neutrophils and were also capable of blocking porcine transferrin from binding to TbpB. Surprisingly, none of the pig or mice antisera from animals immunized with TbpB-derived antigens mixed with Montanide IMS 2215 VG PR adjuvant were able to activate the classical complement pathway (CCP). In contrast, antisera from mice immunized with TbpB-derived antigens adjuvanted with Freund’s adjuvants or Montanide Gel 01 were able to activate the CCP and kill *H. parasuis*. Our results demonstrate that the type of adjuvant can modulate the functional response induced by TbpB-derived antigens. Based on these results, we propose that a properly formulated TbpB-based vaccine may elicit a functional protective antibody response with broad cross-reactivity against heterologous strains of *H. parasuis*.

## Introduction

*Haemophilus parasuis* is a Gram negative, host-specific bacteria commonly found in the upper respiratory tract of conventionally raised pigs. This organism is the etiological agent of Glässer Disease (GD), a systemic inflammatory infection characterized by polyserositis, arthritis and meningitis^1^. Worldwide outbreaks of GD are on the rise and the economic losses associated with it are of major concern to swine producers^2^.

There are at least 15 antigenically distinct serovars of *H. parasuis* and current vaccines formulated with inactivated strains are capable of inducing limited heterologous protection^3, 4^. To overcome the lack of a broadly effective vaccine for *H. parasuis*, management practices and antibiotics are used to reduce GD outbreaks. However, because of consumer demands for antibiotic-free animal products, public health issues, and the growing prevalence of antibiotic-resistant strains, the use of antibiotics may be banned or limited in the future. Thus, an effective vaccine able to induce cross-protection against all heterologous strains is urgently required.

*H. parasuis*, *Actinobacillus pleuropneumoniae* and *A. suis* possess surface receptors that enable them to acquire iron from the host iron-binding protein transferrin (Tf)^5–7^. The surface receptor is comprised of an integral outer membrane protein, Tf binding protein A (TbpA), and a membrane-anchored surface lipoprotein, Tf binding protein B (TbpB). These surface receptors arose in bacteria adapted to the upper respiratory and genitourinary tracts^8^ to acquire iron from Tf on the mucosal surface^9^ and the resulting interplay between host and pathogen has led to the strict host specificity for Tf observed in these pathogens^10^. The ability to acquire iron directly from Tf enables these bacteria to proliferate on the mucosal surface independently from their neighbors and access a readily available iron source during invasive infection. *A. pleuropneumoniae* lacking either TbpB or TbpA are incapable of causing respiratory infections in pigs, suggesting that this receptor complex is required for survival on the mucosal surface of the lower respiratory tract and in the pathogenesis of infection^11^.

The importance of TbpA and TbpB in survival and disease causation prompted efforts at exploring their utility as vaccine antigens against infection by *H. parasuis* ^12, 13^. Detailed characterization of TbpBs from porcine pathogens and their interaction with porcine Tf (pTf)^14–16^ provided the opportunity to rationally engineer mutant proteins defective in binding pTf that were shown to provide superior protection against infection by *H*. *parasuis* ^17^. Follow up experiments suggested that the primary mechanism for superior protection induced by the mutant TbpB was a stronger T helper 2 response^18^. Analysis of the sequence and structural diversity of TbpBs from *H. parasuis, A. pleuropneumoniae* and *A. suis* revealed that this diversity was not associated with geographic region or species and that immunization with intact TbpB generated a substantially greater cross-reactive antibody response compared to the N-lobe, correlating with relative sequence and structural diversity^19^. The aim of this study was to evaluate whether these findings translate into functional antibody responses that could provide a foundation for developing broadly cross-protective vaccines.

## Results

### Immunogenicity of TbpB-derived antigens and cross-reactivity of pig antisera

The mutation of either one of two residues (Y167 or W176) located near to each other on the binding surface of the TbpB N lobe from a serovar 5 (SV5) strain of *H. parasuis* (PDB ID 4O4U and PDB 404X) resulted in a greater that 800-fold drop in binding affinity to pTf^17^, thus these mutant proteins were selected for immunological analyses. Purified preparations of intact wild-type TbpB, intact mutant TbpB (TbpB-M: W176A), and the lobes derived from the mutant TbpB (TbpB-Nm and TbpB-C) (Supplementary Fig. S1) were used to immunize pigs. All recombinant antigens were immunogenic and produced a sufficient serum IgG response after the second immunization (day 35, Supplementary Fig. S2) for cross-reactivity analysis. Amongst the TbpB-derived antigens, the mutant N-lobe (TbpB-Nm) was the least immunogenic.

**Figure 1:**
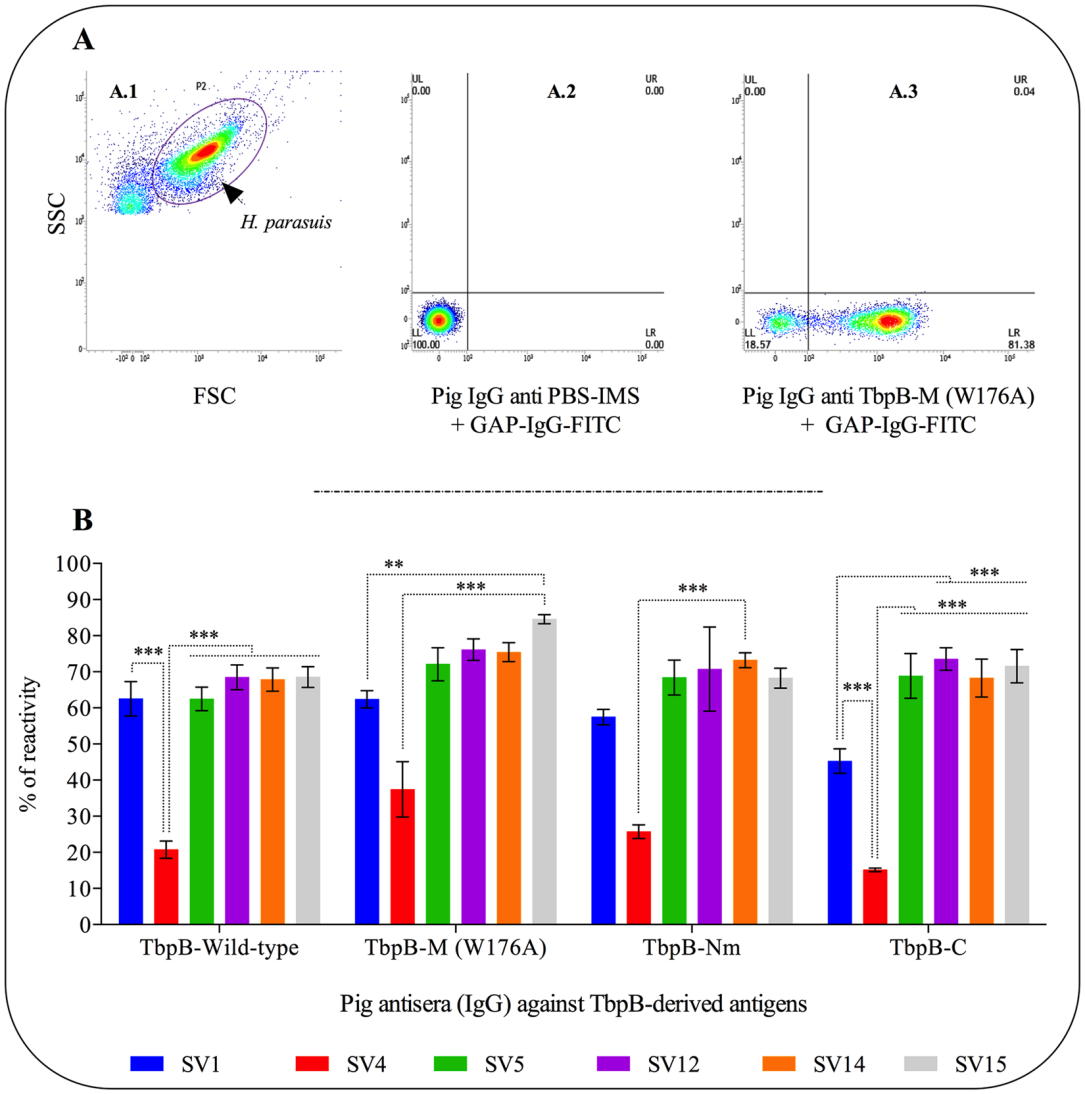
Cross-reactivity profile of pig antibodies (generated against wild-type TbpB, TbpB-M (W176A), TbpB-Nm and TbpB-C) against 6 reference strains of *H. parasuis*. (**A**) Flow cytometry analysis strategy. A.1: Flow cytometric Density Plot (hybrid) of *H. parasuis* (SV15) using side-side scatter (SSC) versus forward-side scatter (FSC) parameters (P2: gate of study). A.2: *H. parasuis* labeled with serum from piglet inoculated with PBS-IMS. The secondary antibody was goat-anti pig IgG FITC labelled (GAP-IgG-FITC) (negative control). A.3: bacteria labeled with serum from a piglet inoculated with TbpB-M, followed by GAP-IgG-FITC. (**B**) Cross-reactivity profile represented as the percentage of the bacterial population recognized by specific pig antibodies. The recognition capacity of each antiserum against 6 reference serovars of *H. parasuis* was statistically compared. Significant differences are represented by asterisks (**p<0.01 and ** p<0.01). Data are expressed as the mean ± SEM.

**Figure 2:**
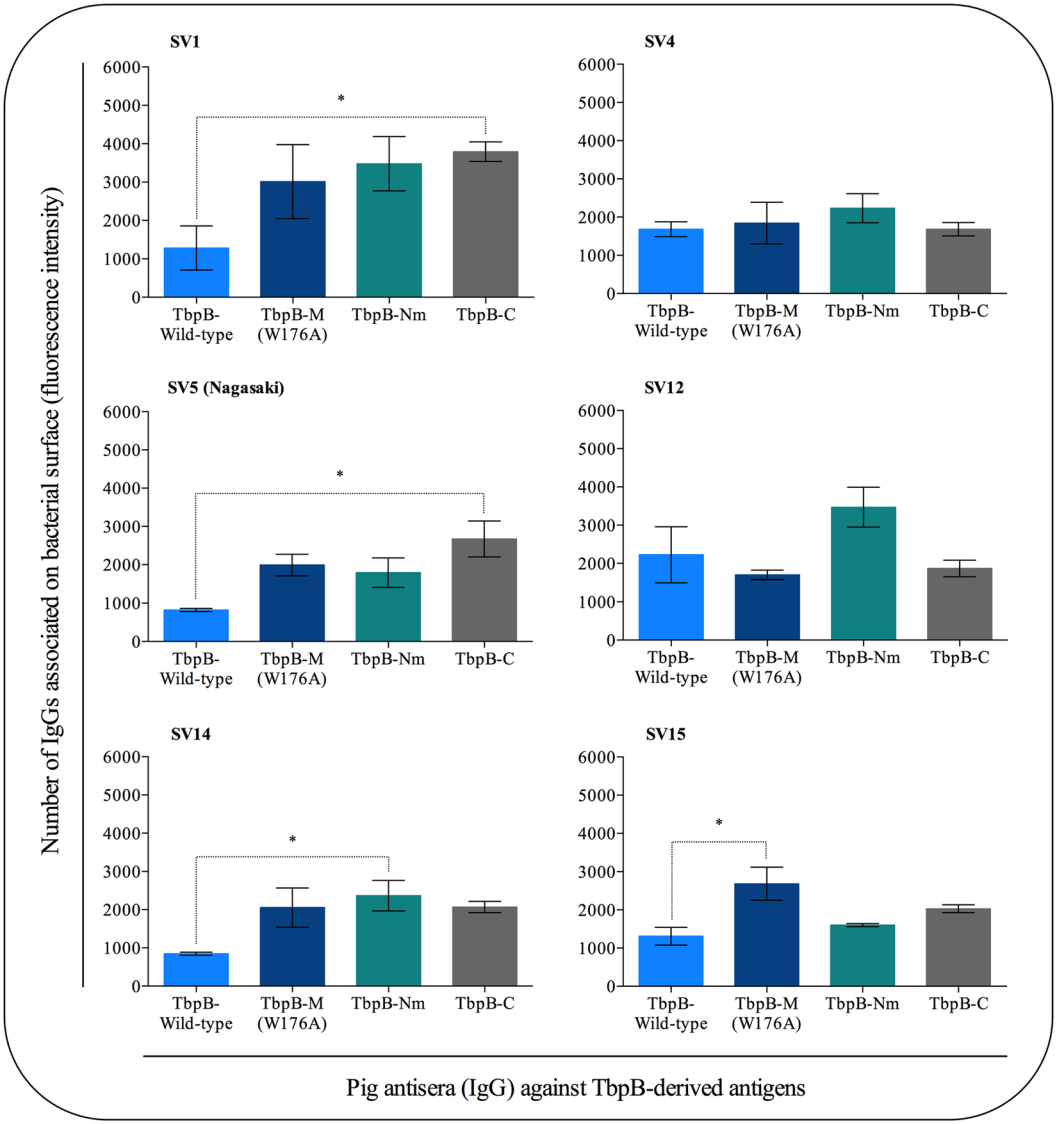
Antigenic analysis of antibodies relative to their binding capacity on the *H. parasuis* surface. The relative number of IgGs associated on surface of 6 reference stains of *H. parasuis* was estimated by the specific fluorescence intensity. The statistical comparison was performed amongst the antisera on each strain. Significant differences are represented by asterisk (*p < 0.05). Bars data are expressed as the mean ± SEM.

The antisera obtained after the second immunization were heated to inactivate endogenous complement and tested for their ability to bind to TbpB variants on the surface of six reference strains of *H. parasuis* belonging to serovars 1, 4, 5, 12, 14 and 15 by fluorescent activated cell sorting (FACS) analysis (Fig. 1). The selected cells (Fig. 1, A.1) were analyzed for the proportion of cells labeled with FITC-labeled anti-pig IgG with the antisera from pigs immunized with adjuvant alone having no detectable binding (Fig. 1, A.2). The number of labeled (right lower quadrant, Fig. 1, A.3) and non-labeled cells (left lower quadrant) were used to determine the proportion of labeled cells (% of reactivity, Fig. 1B) for all of the combinations of sera and representative strains. The mean fluorescent intensity of the labeled cells is illustrated in Fig. 2.

The results showing that all of the antisera preparations labeled a smaller proportion of the SV4 cells (Fig. 1B) was unexpected, and suggests that the lower binding is either due to variation in TbpB expression or accessibility in this strain, phenomena that could be affecting the SV1 cells less dramatically. For interpretation of the cross-reactivity of the sera (Fig. 1B), it is important to recognize that there is considerable sequence diversity of the TbpBs that is independent of serovar^19^ and that relatively high levels of Tf in sera that could compete for epitopes in the binding region. Notably there are cross-reactive epitopes present on both the N-lobe and C-lobe of TbpB expressed on the surface of different *H. parasuis* strains (Fig. 1B and Fig. 2) recognized by antibodies induced by immunizing with individual sub-lobes of TbpB SV5. The trend for lower fluorescence intensity (correlating to relative number of bound IgG) of antisera against wild-type TbpB than against the mutant TbpB in serovar 1, 5, 14 and 15 strains (Fig. 2) could be a reflection of the enhanced antibody recognition of cross-reactive epitopes in or near the binding region capable of competing with the Tf in the sera. Notably, this difference was only statistically significant for the serovar 15 strain.

### Antibody mediated phagocytosis

**Figure 3:**
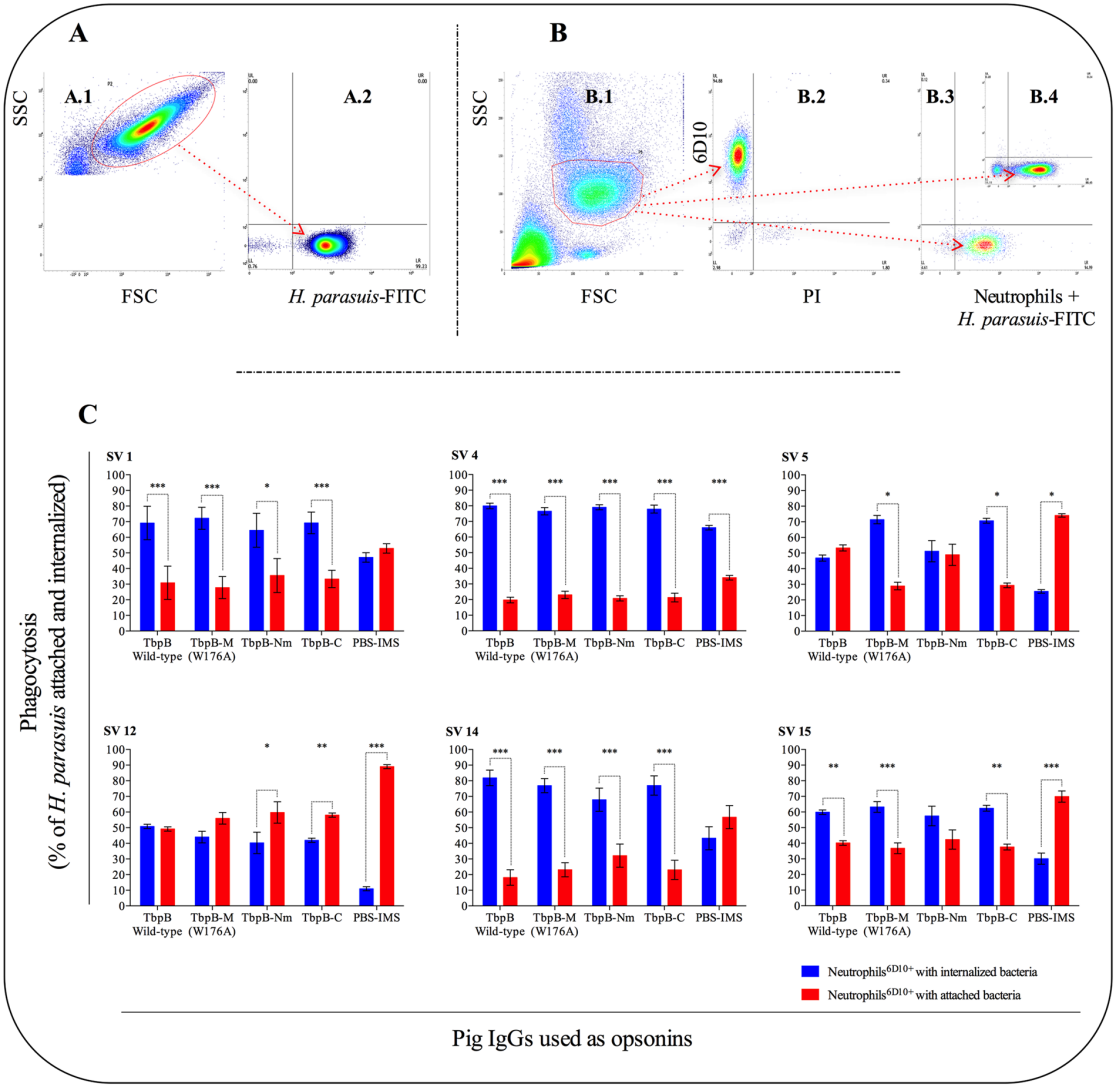
Flow cytometric analysis of pig neutrophil phagocytosis of live opsonized and non-opsonized *H. parasuis*. (**A**) Analysis of the labeling process of *H. parasuis* with FITC. A.1: setting the region (P2) of *H. parasuis* study; A.2: detection of *H. parasuis* labeled with FITC (green fluorescence) at the lower right corner, which represents approximately 99% of bacteria from the P2 region. (**B**) Phagocytosis analysis. B.1: definition of the P5 region for the study of neutrophils based on the FSC and SSC parameters; B.2: demonstration of neutrophils immune-staining with monoclonal antibody mAb 6D10 + rat anti-mouse biotin-labeled IgG2a + SA-APC (∼94% of the cells at the P5 region were neutrophils); B.3 and B.4 neutrophils 15 min after phagocytosis. The cell population at the lower right corner indicate the percentage of neutrophils from the P5 region with associated (at the surface or internalized) *H. parasuis* SV14 and SV5 strains respectively (Table 1). (**C**) Panel demonstrating phagocytosis of serovars 1, 4, 5, 12, 14 and 15 non-opsonized or opsonized with swine antibodies to wild-type TbpB, TbpB-M (W176A), TbpB-Nm or TbpB-C. The red bars represent the percentage of neutrophils with ingested bacteria (after extracellular FITC quenching), and the blue bars represent the percentage of neutrophils with surface adhered bacteria (total neutrophils associated bacteria – percentage of neutrophils FITC positive after quenching). The percentage of adhered and ingested bacteria after the opsonization of each antiserum was statistically compared. Significant differences are represented by asterisks (*p < 0.05, **p < 0.01 and *** p< 0.001). Bars data are expressed as the mean ± SEM.

To evaluate whether the anti-TbpB antibodies would facilitate opsonophagocytosis by neutrophils, a phagocytosis assay with fluorescently labeled *H. parasuis* strains was performed (Fig. 3). Six representative strains of *H. parasuis* were FITC-labeled (Fig. 3A), opsonized with the indicated antibody preparation and mixed with a preparation of peripheral blood neutrophils (Fig. 3, B.1) that was shown to be >94% neutrophils based on labeling with the 6D10 mAb (Fig. 3, B.2). Figure 3, B.3 and B.4 illustrate the distribution of neutrophils with FITC-labeled SV14 or SV5 *H. parasuis*, respectively. The proportion of *H. parasuis* cells internalized by the neutrophils (Fig. 3C) was determined by treatment with trypan blue to quench the fluorescence of extracellular *H. parasuis*. Bacteria exposed to sera from piglets treated with adjuvant alone (PBS-IMS) served as a control for bacteria internalized in the absence of anti-TbpB antibody-dependent opsonization (Fig. 3C). The proportion of internalized bacteria generally increased in the presence of opsonizing antibody against TbpB but notably was minimal in the SV4 strain that had an intrinsically high level of internalization in the absence of TbpB-specific antibody.

There was strain-to-strain variability in the internalization process by neutrophils, with the SV12 strain showing the lowest level of internalization but the greatest dependence on the presence of the opsonizing anti-TbpB antibodies. Nevertheless, the overall results demonstrate that antibodies directed against either of the individual lobes were effective at facilitating internalization by neutrophils and generally were equivalent to antibody directed against both lobes under these assay conditions. There was also strain-to-strain variability in the proportion of neutrophils that were associated with the labeled bacteria (Table 1) and the number of bacteria associated with the neutrophils (Table 2). The proportion of neutrophils with associated bacteria generally increased significantly in the presence of sera obtained from piglets immunized with antigens derived from TbpB. However, there was an insignificant increase with *H. parasuis* SV4 (Table 1). The type of TbpB-derived specific antibodies also affected the ability of neutrophils to uptake opsonized bacteria and in general, the highest fluorescence was observed in neutrophils with bacteria opsonized by anti-TbpB-M antibodies (Table 2), with the exception of *H. parasuis* belonging to SV15.

**Table 1:** Opsonizing effect of sera raised against wild-type TbpB (TbpB-I), W176A mutant TbpB (TbpB-M), mutant TbpB N lobe (TbpB-Nm) and TbpB C lobe (TbpB-C) towards six virulent strains of *H. parasuis*. Numbers in bold font indicates the highest percentage of neutrophils with bacteria associated (attached and phagocytosed). Different letters (a, b or c)  indicates statistically different results (p < 0.05) within the column.

Antiserum	% of neutrophils with associated bacteria after 15 minutes of phagocytosis					
	SV1 (N°4)	SV4 (SW124)	SV5 (Nagasaki)	SV12 (H425)	SV14 (84-22113)	SV15 (84-15995)
TbpB-I	67.6 ± 4.0^a^	39.9 ± 1.9^a^	70.0 ± 3.0^ab^	**62.8** **±** **0.7** ^a^	89.3 ± 1.5^a^	54.3 ± 4.7^a^
TbpB-M	**67.0** **±** **2.3** ^a^	**43.1** **±** **2.9** ^a^	78.4 ± 4.2^bc^	56.0 ± 2.7^ab^	87.8 ± 1.3^a^	**63.5** **±** **2.5** ^a^
TbpB-Nm	56.0 ± 4.5^ab^	43.1 ± 5.7^a^	67.0 ± 5.8^ab^	49.2 ± 3.2^b^	83.8 ± 3.0^a^	54.5 ± 6.5^a^
TbpB-C	62.1 ± 2.9^ab^	38.9 ± 1.1^a^	**86.2** **±** **1.0** ^c^	52.2 ± 1.6^b^	**91.3** **±** **1.3** ^a^	57.4 ± 3.0^a^
PBS-IMS	50.5 ± 2.0^b^	37.8 ± 1.3^a^	54.9 ± 1.3^a^	26.4 ± 1.2^c^	56.3 ± 4.4^b^	32.1 ± 2.5^b^

**Table 2:** Indirect quantification of the number of neutrophil-associated bacteria determined as the mean fluorescence intensity emitted by the FITC-labeled bacteria after phagocytosis. The numbers in bold highlights the antibodies that mediated higher bacterial attachment and phagocytosis by neutrophils. Different letters (a, b or c) indicates statistically different results (p < 0.05) within the  column.

Antiserum	Mean of fluorescence intensity of neutrophils after 15 minutes of phagocytosis					
	SV1 (N°4)	SV4 (SW124)	SV5 (Nagasaki)	SV12 (H425)	SV14 (84-22113)	SV15 (84-15995)
TbpB-I	1,779 ± 45^d^	475 ± 20^a^	5,067 ± 352^b^	1,194 ± 37^b^	868 ± 68^b^	541 ± 19^ab^
TbpB-M	**2,101** ** ±** **77** ^c^	**519** **±** **2** **6** ^a^	**6,798** ** ±** **128** ^b^	**1,297** **±** **27** ^b^	**976** **±** **40** ^ab^	548 ± 28^ab^
TbpB-Nm	1,353 ± 29^b^	467 ± 25^a^	5,005 ± 343^b^	1,035 ± 101^ab^	809 ± 76^ab^	513 ± 41^ab^
TbpB-C	1,461 ± 35^b^	514 ± 44^a^	6,554 ± 204^b^	1,066 ± 21^ab^	942 ± 32^b^	**575** **±** **18** ^b^
PBS-IMS	380 ± 10^a^	419 ± 12^a^	2,655 ± 359^a^	857 ± 82^a^	668 ± 42^a^	425 ± 23^a^

### Complement activation

Using the complement depletion assay we were unable to detect complement activation using piglet sera directed against any of the antigens [wild-type TbpB, TbpB-M (W176A), TbpB-Nm or TbpB-C]. These preliminary results led us to hypothesize that the subclass of antibodies induced by immunization with TbpB adjuvanted with Montanide IMS were unable to activate complement.

To test our hypothesis, we compared TbpB vaccine compositions formulated with our original adjuvant, Montanide IMS 2215 VG PR, or either Freund’s adjuvant or Montanide Gel 01 PR in a mouse immunization protocol and evaluated the resulting antisera for complement activation (Table 3). The sera from mice immunized with antigen mixed with Montanide Gel 01 PR or Freund’s adjuvant had a similar complement activation profile toward the 6 virulent serovars of *H. parasuis* tested. With the exception of antibodies generated against wild-type TbpB, all antibodies were capable of inducing complete (100%) complement activation against reference strains belonging to SV1, SV4, SV5, SV12, SV14 and SV15. Anti wild-type TbpB antibodies induced using Freund’s and Montanide Gel 01 PR adjuvants partially activated complement against SV 1 (50% and 20%, respectively), SV 4 (80% both) and SV 5 (50% both) (Table 3). Similar to the results obtained with the pig antisera, none of the mice immunized with the antigens mixed with Montanide IMS 2215 VG PR were capable of activating complement in this depletion assay.

**Table 3:** Activation of classical pathway of complement system by sera against TbpBs produced in mice. Antibodies from mice immunized with wild-type TbpB, TbpB-M, TbpB-C and TbpB-Nm adjuvanted with Montanide IMS 2215 VG PR, Montanide Gel 01 or Freund’s adjuvant were tested against 6 virulent serovars of *H. parasuis*. The capacity of these sera to activate the complement system is represented as percentage of activation.

Adjuvant	Reference serovar	Antigens classical pathway activation of complement system (%)			
		TbpB-I	TbpB-M	TbpB-C	TbpB-Nm
Montanide IMS 2215 VG PR	1	0	0	0	0
	4	0	0	0	0
	5	0	0	0	0
	12	0	0	0	0
	14	0	0	0	0
	15	0	0	0	0
Montanide Gel 01 PR	1	20	100	100	100
	4	80	100	100	100
	5	50	100	100	100
	12	0	100	100	100
	14	0	100	100	100
	15	0	100	100	100
Freund’s adjuvant	1	50	100	100	100
	4	80	100	100	100
	5	50	100	100	100
	12	0	100	100	100
	14	0	100	100	100
	15	0	100	100	100

To determine whether the mouse immunoglobulin isotype induced by the Montanide IMS 2215 VG PR differed from the isotype elicited by Freund’s adjuvant or Montanide Gel 01 PR as a potential reason for the inability to activate complement, we evaluated the immunoglobulin isotype distribution in the mouse sera. The results showed that both Montanide adjuvants and Freund’s adjuvant induced similar subclass distribution of immunoglobulins, however, the magnitude of the IgG1 and IgG2a titres were approximately 16 times higher in the animals immunized with Gel 01 PR than in those immunized with IMS 2215 VG PR (Fig. S3). Animals immunized with Freund based adjuvants had the highest titres for every immunoglobulin isotype.

**Figure 4:**
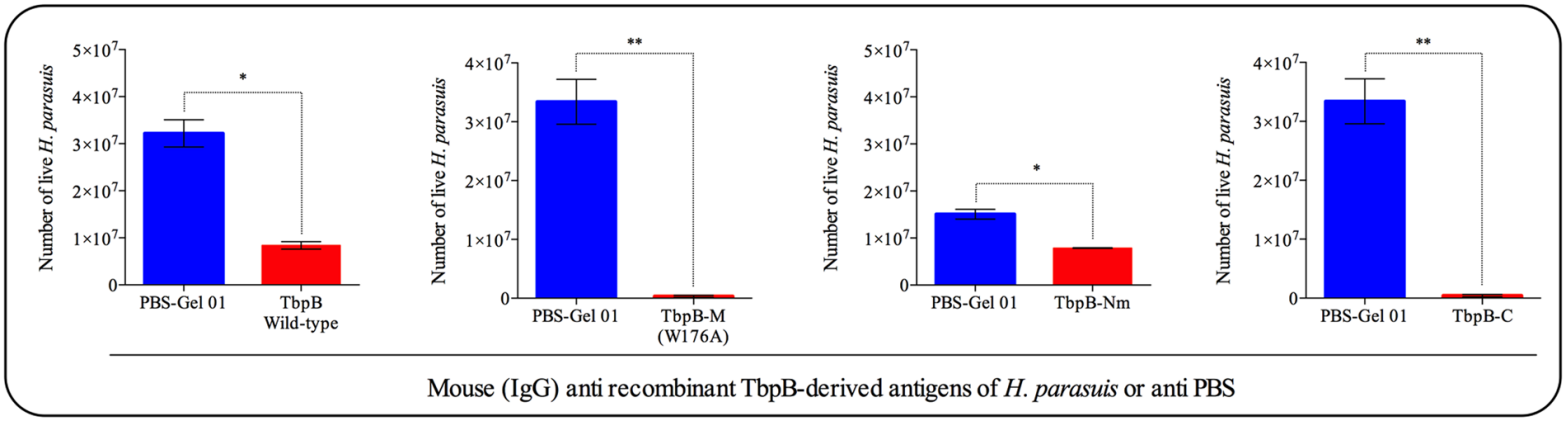
Classical complement activation and killing pathways of *H. parasuis* Nagasaki strain by mouse anti-TbpB antibodies. Antibodies against wild-type TbpB, TbpB-M (W176A), TbpB-Nm and TbpB-C killed ~73%, ~98%, ~45% and ~98% of the bacterial population in the assay, respectively. The statistical comparison was performed between bacteria treated with antiserum against each TbpB derived antigen and control serum (mouse anti PBS-Gel 01 IgG). Significant differences are represented by asterisks (*p < 0.05 and **p < 0.01). Bars data are expressed as the mean ± SEM.

In order to test the ability of immune sera to induce killing by antibody mediated complement activation, the sera from mice immunized with the different recombinant antigens adjuvanted with Montanide Gel 01 PR were tested in a serum bactericidal assay (Fig. 4). All of the mouse sera reduced (p < 0.01) the number of live *H. parasuis* SV5 (Nagasaki strain) after incubation with exogenous complement. Antibodies to TbpB-M (W176A) and TbpB-C reduced bacterial viability by 98%, while anti wild-type TbpB and anti-TbpB-Nm killed approximately 73% and 45% of the *H. parasuis*, respectively.

### Transferrin binding site blocking with antibodies induced by mutant TbpBs

**Figure 5:**
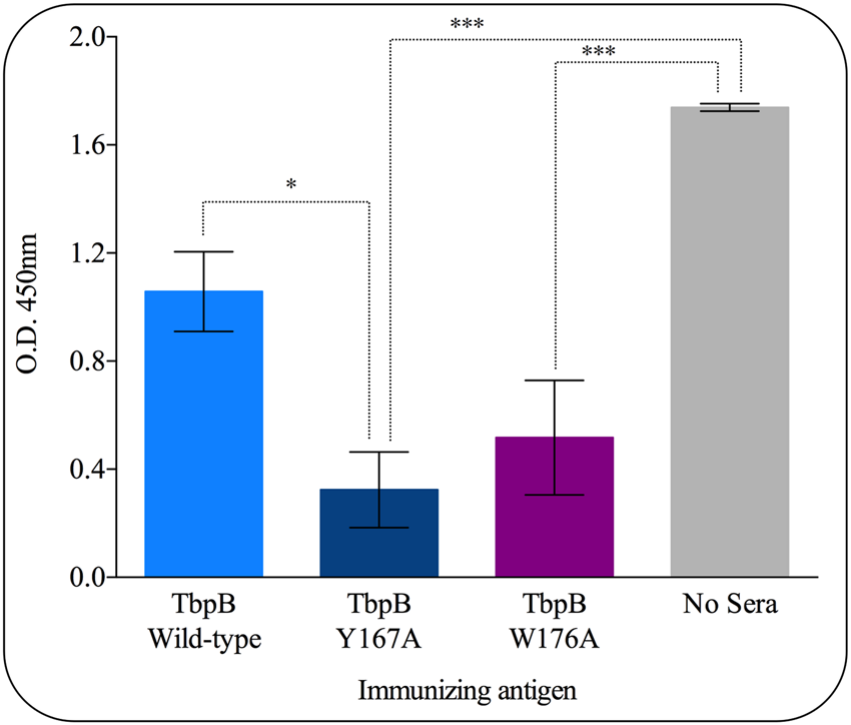
Ability of sera to block Tf binding to TbpB *in vitro*. Wells coated with Nagasaki strain TbpB were treated with purified serum IgG from pigs immunized with wild-type TbpB, Y167A TbpB or W176A TbpB. HRP-labeled Tf was added and the absorbance (450 nm) was measured. Purified serum IgG from pigs were assayed individually in triplicate and then averaged. Data show the mean of pigs per group ± SEM. Significant differences between the antigens are represented by asterisks (*p < 0.05 and ***p < 0.001) as determined by a one-way ANOVA with post-hoc analysis done with Tukey’s multiple comparisons test.

To test the hypothesis that the Tf binding surface of native TbpB is blocked from the immune system by endogenous host Tf during systemic immunization and that non-binding TbpB mutants will therefore elicit antibody directed at this surface that are capable of blocking Tf, pigs were immunized with one of either wild-type TbpB or with one of two different non-binding TbpB mutants (Y167A or W176A). The respective sera were used to compare the ability to block the binding of pTf to TbpB *in vitro*. Preliminary experiments with whole sera did not reveal any significant differences due to interference by endogenous pTf in the serum samples. Therefore, purified serum IgG was used in a competitive binding assay. As illustrated in Fig. 5, there was a significant decrease (p < 0.001) in pTf binding for sera from either mutant TbpB immunization compared to control as well as a significant decrease (p < 0.05) of pTf binding by sera from pigs immunized with Y167A TbpB compared to sera from pigs immunized with wild-type TbpB.

### Protective response induced by TbpB or TbpB lobes

**Figure 6:**
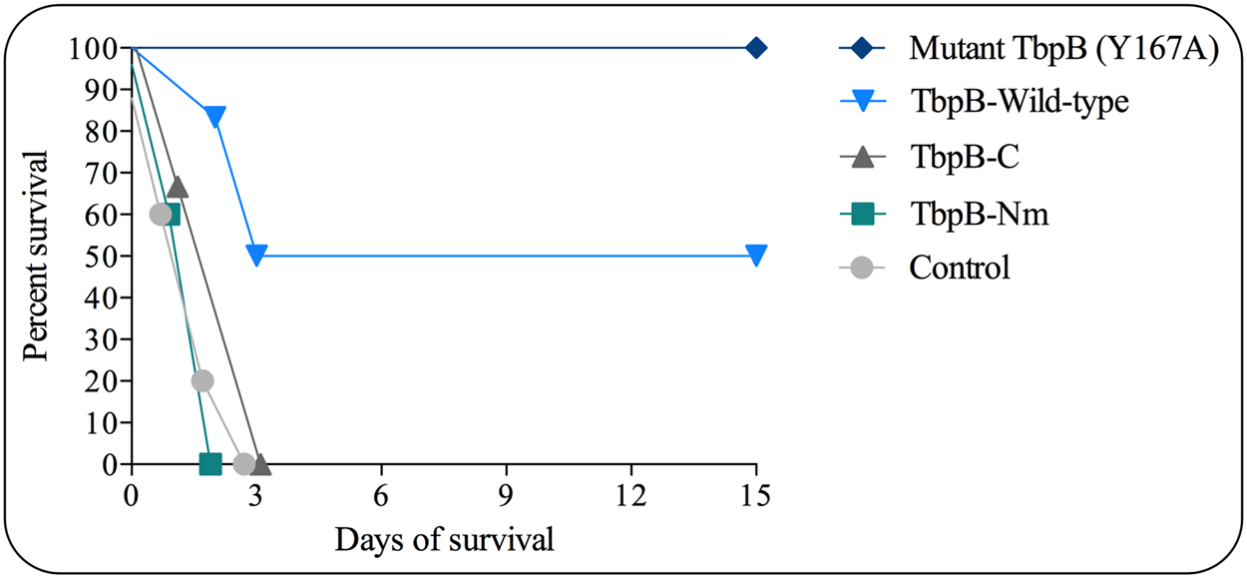
Comparative ability of TbpB or its lobes to induce a protective immune response against *H. parasuis* infection. Groups of pigs were immunized with adjuvant alone (control), Y167A mutant TbpB, wild-type TbpB, TbpB-C or TbpB-Nm of *H. parasuis* serovar 5. The pigs were challenged by intratracheal inoculation with 10^8^ colony forming units (CFU) of the same strain and they were monitored for clinical signs and symptoms throughout the length of the experiment (14 days), after which pigs were examined by necropsy. The data presented in this figure are derived of the experiment previously published by Frandoloso, *et al*.^17^.

Since TbpB is a lipoprotein anchored to the outer membrane by the N-terminal anchor peptide region, both lobes of the protein may be accessible to antibody and other immune effector mechanisms. Thus, it is an important question as to whether less variable regions of the protein, primarily located in the C-lobe^19^, could be used to induce a more cross-protective response. Groups of pigs (6 per antigen) were immunized with the intact wild-type TbpB, Y167A mutant TbpB or with its constituent mutant N-lobe or C-lobe and were subjected to intratracheal challenge with 10^8^ CFU of *H. parasuis* Nagasaki strain, along with control pigs (6 animals) treated with PBS + Montanide IMS 2215 VG PR. In contrast to the group immunized with Y167A mutant TbpB, in which 100% of pigs survived until the end of the experiment, 3 pigs immunized with wild-type TbpB survived until the end of the experiment (50%). None of the pigs immunized with the C-lobe or mutant N-lobe survived significantly longer that the control animals (all pigs immunized with adjuvant alone or the TbpB C-lobe reached endpoint by day 3, while all pigs immunized with the TbpB mutant N-lobe reached endpoint by day 2). Survival time of pigs immunized with intact TbpB was significantly higher than those of C-lobe, mutant N-lobe and control pigs (p < 0.01 in all cases) (Fig. 6). Pigs immunized with wild-type TbpB had fewer systemic locations with recoverable *H. parasuis* at the time of necropsy compared to pigs immunized with either TbpB sub-lobes or adjuvant alone (Table 4).

**Table 4:** Bacterial recovery on chocolate blood agar in different treatment groups infected with the *H. parasuis* Nagasaki strain. Colonies were verified by PCR as *H. parasuis* ^32^.

Tissues	Number of pigs positive for *H. parasuis* in				
	Y167A Mutant TbpB*	Wild-type TbpB*	TbpB-C	TbpB-Nm^a^	Control*^b^
Lung	0	3	5	4	4
Spleen	0	2	1	4	4
Brain	0	2	0	3	2
Abdominal cavity	0	4	4	3	5
Hock joints	0	2	5	2	4
Heart blood	0	3	4	5	4

^a^The recovery rate in the six locations sampled was significantly higher than those of mutant TbpB (Y167A) and intact wild-type TbpB (p < 0.05); ^b^The recovery rate in the six locations sampled was significantly higher than those of the remaining three groups compared (p < 0.01). *Data from published study^17^.

## Discussion

The pathogenesis of GD can be viewed to involve three main stages; (i) transmission and colonization of the upper respiratory tract by *H. parasuis*, (ii) proliferation and spread into the lower respiratory tract, and (iii) breach of the mucosal barrier and systemic spread of the bacteria. The observation that parenteral administration of conjugate capsular vaccines not only prevented infection by the targeted bacteria but effectively eliminated them from the upper respiratory tract of humans^20, 21^ indicates that prevention of colonization can be accomplished even without administration of vaccines through the mucosal route.

Although it has not been experimentally addressed, it is possible that vaccines derived from intact bacteria could be affecting colonization and therefore disrupting transmission. However, similar to the conjugate capsular vaccines, whole-cell vaccines tend to be strain specific and may be overcome by horizontal gene exchange and acquisition of alternate dominant surface antigens^22^. Therefore, in pathogens such as *H. parasuis* that have variable and exchangeable surface antigens, whole-cell vaccines formulated with one or more serovars demonstrate variable degrees of protection^3, 4, 23, 24^ which might be limited by the type of adjuvant used^24^. The demonstration that antigenic variants of the Tf receptor proteins are distributed amongst three porcine pathogens, *H. parasuis*, *A. pleuropneumoniae* and *A. suis* ^19^, indicates that reservoirs of antigenic variants outside of *H. parasuis* must be considered during the development of broadly cross-protective and long-lasting vaccines.

The importance of Tf receptors for survival on the mucosal surface^11, 25^ makes them potential targets to intercept *H. parasuis* at the colonization stage, particularly if antigens capable of inducing a broadly cross-protective response are developed. The experimental challenge model for *H. parasuis* infection^12, 13, 17, 18^ involves direct intratracheal administration of the bacteria and thus addresses the two subsequent phases of pathogenesis after colonization. As a consequence, it is not readily apparent whether the enhanced protection induced by a non-binding mutant of TbpB^17^ and the lack of protection observed with the individual lobes (Fig. 6) are primarily due to mechanisms occurring on the lower respiratory tract or during the subsequent invasive systemic infection. That we had to purify IgG from pig serum in order to demonstrate the enhanced blocking by serum against mutant TbpBs (Fig. 5) suggests that the impact on iron acquisition may be most important on the upper and lower respiratory tract, where the levels of Tf on the mucosal surface are dramatically lower than in serum.

The final stage of the pathogenesis of *H. parasuis* infection involves crossing the epithelial cell layer of the mucosal surface and entering the blood or lymphatic stream for systemic spread of disease. Since neutrophils are the first line of defense against extra-cellular bacterial pathogens peripheral blood neutrophils were used to evaluate the functional attributes of the antibodies induced with the TbpB-derived antigens. To address the potential cross-protective properties of the immune response we evaluated the heterologous recognition of representative *H. parasuis* strains from serovars 1, 4, 5, 12, 14 and 15, all of which are known to trigger GD^26^. Using FACS analysis, we were able to demonstrate binding of IgG directed against the TbpB-derived antigens from the Nagasaki strain by all of the representative strains analyzed (Fig. 1B), indicating that there are conserved or shared epitopes amongst the variant TbpBs tested here. The bound antibodies were able to facilitate internalization of the representative strains by neutrophils (Fig. 3) and antibodies directed against both lobes were able to facilitate opsonophagocytosis, suggesting that this property was not responsible for the failure of individual lobes to induce protection against intratracheal challenge by *H. parasuis* (Fig. 6).

None of the pig antisera mediated complement activation *in vitro*. As the type of immune response and immunoglobulin class might be related to the type of adjuvant used in the immunization^27^, we hypothesized that the Montanide IMS 2215 VG PR adjuvant could have induced an IgG subclass with reduced capacity to induce complement activation, such as IgG1^28^, induced mostly by IL-10^29^. We previously demonstrated that the use of Montanide IMS 2215 VG PR for immunization in pigs with TbpB increased the transcription of Th2 cytokines, including IL-10 ^18^. Thus, to explore the role of adjuvants on the type and functionality of the antibodies produced against the recombinant antigens, mice were immunized with antigens mixed with three different adjuvants (Montanide IMS 2215 VG PR, Montanide Gel 01 PR and Freund adjuvant). Montanide IMS 2215 VG PR was uniquely unable to activate complement in this experiment (Table 3). The distribution of IgG subclasses induced by Montanide IMS 2215 VG PR and Montanide Gel 01 PR were similar, however the titres of IgG1 and IgG2a was higher with Montanide Gel 01 PR (Fig. S3). This observation appears to suggest a direct dependence between the magnitude of these titres and the ability to activate the complement system and contradicts our initial hypothesis relating to the immunoglobulin subclass and complement activation. This may also explain the failure to achieve protection with the N-lobe or C-lobe in the challenge experiment in pigs (Fig. 6). Since the adjuvant modulation observed in mice could not be fully translated to the pig model, future experiments are needed to address this question.

In conclusion, *H. parasuis* TbpB recombinant proteins are capable of inducing antibodies that recognize variant TbpBs across a series of representative strains and conserved epitopes are distributed on both TbpB lobes. These antibodies are capable of mediating internalization of bacteria by neutrophils. As adjuvant appear to dramatically influence the development of complement activating antibodies, the choice of the correct adjuvant may facilitate the induction of antibodies able to mediate complement activation and therefore increased bacterial killing. Since immunization with non-binding mutants of TbpB appears to elicit antibodies capable of inhibiting iron acquisition on the mucosal surface, the prospects of developing broadly cross-protective vaccines with a selection of mutant TbpBs or their individual lobes are encouraging.

## Material and Methods

### Expression and purification of recombinant TbpB

The recombinant proteins were expressed and purified essentially as previously described^14^. The gene encoding wild-type TbpB (TbpB^20–537aa^) from a *H. parasuis*, serovar 5 strain was cloned into an expression vector and two different non-binding mutants were prepared, Y167A and W176A^17^. The mutant Y167A gene was then used as a template for amplifying the region encoding the subdomains (lobes) N (TbpB-Nm^20–290aa^) and C (TbpB-C^291–537aa)^. These amplified fragments were cloned into the pET20a expression vector resulting in gene encoding a maltose binding protein (MBP)-fusion protein containing a N-terminal polyhistidine tag and a Tobacco Etch Virus (TEV) protease cut site located immediately prior to the TbpB coding region. The expression of the fusion protein was induced by 0.1 mM isopropyl β-D-1-thiogalactopyranoside (IPTG) (Sigma Aldrich, USA) and the fusion protein purified with a Ni-NTA column (HisTrap™HP, GE Healthcare, USA) followed by cleavage with TEV protease and purification of the TbpB derived protein by ionic exchange (Sepharose-Q, GE, Germany).

### Experimental vaccine formulation and swine immunization protocol

Thirty mixed-bred (Large White × Landrace) piglets were used in the first experiment. Piglets were deprived from maternal colostrum and kept in a facility room free of specific pathogens (SPF) under biological security level II. Piglets were fed pasteurized cow colostrum and milk followed by commercial feed pellets and were found to be free of anti-*H. parasuis* antibodies. Twenty-eight days after birth the piglets were weighed, tagged and divided into five groups (6 piglets/group). The piglets in each group were then immunized with 200 μg of intact TbpB (wild-type TbpB), mutant TbpB (TbpB-M, W176A), mutant TbpB N lobe (TbpB-Nm) or mutant TbpB C lobe (TbpB-C), respectively, adjuvated with 25% Montanide IMS 2215 VG PR (Seppic, France) in a 2 ml final volume, by the intramuscular route. The fifth, control group was injected with sterile phosphate buffered saline (PBS, pH 7.2) mixed with Montanide 2215 VG PR (PBS-IMS). All piglets were immunized again 21 days later with the same vaccine formulation. Blood samples were collected prior to the first and second immunizations and 14 days after the last shot. The experiment followed the guidelines of the Brazilian College of Animal Experimentation and was approved by the institutional Committee for Ethical Use of Animals (protocol n° 015/2014).

In the second experiment, fifteen six-week old male, castrated pigs were immunized on days 0, 22 and 42 with 100 μg of antigen (wild-type TbpB from *H. parasuis* SV 5 or one of the two non-binding mutants, Y167A or W176A) in sterile PBS adjuvanted with 20% Emulsigen D to a final volume of 2 ml. Pigs were immunized intramuscularly and blood was collected prior to each immunization and again at day 56 and sera were stored at −80 °C until use. This immunization series was completed under protocol AC11-0063 at the University of Calgary Spyhill campus.

Finally, supplementary results from a third experiment partially published by Frandoloso, *et al*.^17^ were included in this study. In addition to the two experimental vaccines already published (based on wild-type TbpB and on Y167A mutant TbpB) another two were evaluated in the same experiment and are presented here. Briefly, twelve colostrum deprived piglets were randomly assigned in two groups of six animals each and immunized with TbpB-Nm or TbpB-C recombinant antigens. The vaccines were formulated with 200 μg of proteins mixed with 25% of Montanide IMS 2215 VG PR in a 2 ml final volume, and administered by the intramuscular route at 28 and 49 days of age. Twenty-five days after the second immunization, all animals were challenged with a lethal dose (10^8^ CFU) of *H. parasuis* Nagasaki strain injected by intratracheal administration. This experiment was conducted in accordance with the guidelines of the University of León Ethical Committee and the Spanish Government.

### Polyclonal mouse antibody production

Fifty micrograms of purified TbpB-I, TbpB-M (W176A), TbpB-Nm or TbpB-C mixed with Freund’s complete adjuvant (FCA) (ratio 1:1.2), Montanide IMS 2215 VG PR (25% v/v) or Montanide Gel 01 PR (15% v/v) (Seppic, France) in 0.5 ml final volume were injected by the intraperitoneal route into two eight-week old Balb/c mice each. For a negative control, PBS was mixed with each adjuvant and injected into two mice each. Twenty-one days later all mice were re-immunized with the same formulation except mice immunized with FCA where the second immunization was carried out using Freund’s incomplete adjuvant. Blood samples were collected by cardiac puncture from mice anesthetized with isoflurane (Cristália, Brazil) at fourteen days after the second immunization. After clotting, serum was separated and frozen at −80 °C until use.

### Cross-reactivity analysis of serum by flow cytometry

Six reference strains of *H. parasuis* (N°4–SV1, SW124–SV4, Nagasaki–SV5, H425–SV12, 84-22113–SV14 and 84-15995–SV15) were cultured on pleuropneumoniae-like organisms broth (Difco, USA) supplemented with 40 μg/ml of nicotinamide adenine dinucleotide (Sigma Aldrich, Brazil), 2.5 mg/ml glucose (Sigma Aldrich, Brazil) and 200 μM of 4,4-dipyridyl (Sigma Aldrich, Brazil). The bacteria were cultured under constant rotation (250 rpm) at 37 °C for 12 h and then washed twice with PBS and counted. Serum samples from all immunized piglets were heat-treated (56 °C, 30 min) and aliquots of 10 μl were incubated with 1 × 10^6^ bacteria for 1 h at 37 °C. The bacteria were washed three times with PBS and coupled antibodies were detected using fluorescein labeled (FITC) goat anti-piglet IgG (AbD Serotec, UK) diluted 1:1000 in PBS with 1% bovine serum albumin (BSA) for 1 h at 37 °C. The bacteria were then washed again three times with PBS and suspended in 200 μl of FACS buffer (PBS + 0.5% BSA, pH 7.2) and analyzed by flow cytometry (BD FACSVerse™ – BD Biosciences, USA) equipped with blue and red laser, and volumetric counter. The bacterial population was characterized according to size (forward-angle scatter – FSC), complexity (side-angle scatter – SSC) and green fluorescent emitted by the FITC. The data were analyzed using BD FACSuite™ software (BD Biosciences, USA).

### Isolation of pig peripheral polymorphonuclear (PMN) leukocytes

Blood samples from two pigs (mixed-bred: Large White × Landrace) were collected using tubes containing EDTA. PMN were isolated using Ficoll-Paque Plus (GE Heathcare, Sweden) following manufacturer’s instructions, and contaminating erythrocytes were lysed using Red Blood Cell (RBC) Lysing Buffer (Sigma Aldrich, Brazil). The purity and cell identity were confirmed by flow cytometry taking into consideration the characteristics of the cells (FSC *vs* SSC) and the expression of the antigen recognized by monoclonal antibody (mAb) clone 6D10 isotype IgG2a (kindly provided by Dr. Javier Domínguez Juncal, Spain) followed by detection with biotin rat anti-mouse IgG2a (clone R19-15, BD Pharmingen™, USA) in the presence of streptavidin allophycocyanin conjugate (SA-APC) (BD Pharmingen™, USA). Cell viability was evaluated by propidium iodide (PI, Invitrogen, Brazil) and was consistently higher than 95%.

### *Haemophilus parasuis* labeling with FITC

*H. parasuis* was labeled as previously described^30^ with minor modifications. All strains were grown in iron-restricted PPLO broth (containing 100 μM of dipyridyl) to reach an optical density (OD) of 0.7 at 600 nm (Nanophotometer, Implen, Germany). Bacteria were then washed three times with PBS, counted by flow cytometry and, for labeling, 1 × 10^9^ bacteria/ml in PBS were incubated with 1 μg of FITC (Sigma Aldrich, Brazil) under agitation (300 rpm) for 30 min at 22 °C. The labeled bacteria were then washed twice with an excess of PBS to remove unbound FITC and then suspended in 10 ml of PBS containing 5% BSA for 15 min. Labeled bacteria were collected by centrifugation (4,000 × *g*, 10 min) and suspended in 1 ml of PBS and kept at 4 °C.

### Antibody-mediated phagocytosis

The phagocytic assay was performed as previously described by Olvera, *et al*.^30^ with minor modifications. All FITC-labeled bacteria used in this study (strain N°4 [SV1], SW124 [SV4], Nagasaki [SV5], H425 [SV 12], 84-22113 [SV 14] and 84-15995 [SV 15]) were incubated (1 × 10^7^ cells) with 100 μl of specific dilution of the polyclonal piglet antibodies [anti wild-type TbpB, TbpB-M (W176A), TbpB-C and PBS diluted 1:128 and anti-TbpB-Nm diluted 1:64] at 37 °C for 45 min under constant agitation (300 rpm). The opsonized bacteria were then washed to remove non-coupled antibodies and incubated with 1 × 10^6^ neutrophil cultures in 96 well plates (TPP, Sweden) at 37 °C in a final volume of 100 μl of RPMI 1640 medium (Invitrogen, Brazil) supplemented with 10% fetal bovine serum (FBS) (Invitrogen, Brazil) and 100 mM of L-glutamine (Sigma Aldrich, Brazil). After 15 min, the plates were incubated on ice, washed three times with PBS + 1% FBS and suspended in 200 μl of PBS + 1% FBS. Cells were then analyzed by flow cytometry and the total number of neutrophil-associated bacteria was estimated by total fluorescence. In addition, intracellular bacteria were calculated after quenching with trypan blue (31 μg/ml; Sigma Aldrich, Brazil)^31^, subtracting from the total positive neutrophils the percentage of FITC positive cells after the addition of quencher.

### Activation of the complement classical pathway and antibody bactericidal activity

The capability of serum from immunized piglets and mice were evaluated for ability to activate the classical pathway of complement system and kill *H. parasuis*. All strains of *H. parasuis* were grown in iron-restricted media, washed twice with CSAB buffer (210 mM triethanolamine, 180 mM citric acid, 10.5 mM MgCl, 1.8 mM CaCl, 1.3 M NaCl, pH 7.4) and counted. Then, 1 × 10^7^ bacteria were mixed with 50 μl of inactivated serum [pig sera against wild-type TbpB, TbpB-M (W176A) and TbpB-C were diluted 1:5 and against TbpB-Nm were diluted 1:2.5 in CSAB. Mice sera against wild-type TbpB, TbpB-M (W176A) were diluted 1:320 and against TbpB-C and TbpB-Nm was diluted 1:160 in the same buffer. The sera titration was conducted by flow cytometry and the dilution selected was those capable to recognized approximately 1 × 10^7^ *H. parasuis* Nagasaki strain], 25 μl of guinea pig complement containing 5 units of C’H50 (see below) in a 96-well plate, and incubated for 1 h at 37 °C. Then, 25 μl of the hemolytic system (HS) comprised of sheep red blood cells (SRBC, 2%) sensitized with rabbit hyper-immune serum was added and incubated for 1 hour at 37 °C. The plates were then centrifuged (250 × *g* for 2 min) and the supernatant were harvested and read at 540 nm for estimating the amount of lysed sensitized (SRBC).

The reagents used in this assay (rabbit anti-SRBC hyper-immune serum and guinea pig complement) were titrated prior to the experiments. The optimal dilution of rabbit anti-SRBC serum was 1:1,500 and the dilution of guinea pig complement giving 50% hemolysis (C’H50) of SRBC was 1:800. Five units of C’H50 were used in the assay. In the complement assay, the following controls were used: (a) positive antibody control [pig hyperimune serum against *E. coli* TOP10 + TOP10 cells + C’ + CSAB buffer] and HS: no hemolysis; (b) C’ control [C’ + CSAB] and HS: 100% hemolysis; (c) serum nonspecific C’ inhibitors [pig serum + C’ + CSAB] and HS: 100% hemolysis; (d) classical activation pathway control [TOP10 cells + C’ + CSAB] and HS: 100% hemolysis; (e) hemolysin control [*H. parasuis* Nagasaki strain + CSAB] and HS: no hemolysis and (f) activation buffer control [CSAB only] and HS; no hemolysis.

The assay was interpreted as follows: the presence of SRBC hemolysis, detected by spectrophotometry (540 nm), indicated that the antibodies were unable to activate the complement system. In contrast, the absence of hemolysis indicated that the complement system was activated and was depleted as consequence of antibodies-antigen complexes developed in the assay.

The bactericidal assay evaluated the capability of the antibodies to kill the bacteria by activating the complement classical pathway. The assay was similar to that described for complement activation. However, the efficacy of mice specific antibodies (generated against TbpB-derived recombinant antigens) + complement system to kill the *H. parasuis* Nagasaki strain was evaluated in this assay. The reaction consisted of *H. parasuis* (1 × 10^6^) + mice serum + C’ + CSAB buffer, which was incubated at 37 °C for 1 h. Then, the non-killed bacteria were detected by culturing on chocolate agar and counted after 24 h of incubation at 37 °C. Negative control consisted of serum from mice inoculated with PBS. Each serum was analyzed in duplicate in two independent assays.

### Mouse immunoglobulin isotyping

To measure TbpB-isotype specific antibody titres induced by the three adjuvants evaluated (IMS 2215, Gel 01 and Freund’s), mouse sera were added to an ELISA plates coated with 1 μg of wild-type TbpB. Mouse antisera were serially diluted in PBST containing 1% of Skim Milk (Sigma-Aldrich) and added to the wells (100 μl) and incubated for one hour at 37 °C. Then, wells were washed 3 times with PBST and rat anti-mouse IgG1, IgG2a, IgG2b, IgG3, IgM and IgA (BD Biosciences, USA) were added to the wells (100 μl of 1:10 diluted Ig, as recommended by the manufacturer) and incubated for one hour at 37 °C. Afterward, wells were washed again and 100 μl of rabbit anti-rat IgG peroxidase conjugated (Sigma Aldrich, Brazil) diluted 1:10,000 was added to the wells and incubated as indicated above, followed by three washes and addition of substrate (3,3, 5,5′- tetramethylbenzidine + 0.06% H_2_O_2_). The plates were then incubated in the dark at 22 °C for 15 minutes and the reaction was stopped by adding 3 N HCl. Plates were read at 450 nm using a Synergy HI plate reader (Bio-Tek, USA). The results were described as endpoint titres which are the reciprocal of the highest dilution that gave a positive OD reading [defined as at least two times greater that the OD values of the negative samples (mouse inoculated with PBS + adjuvant)].

### ELISA analysis

The indirect ELISAs were performed in order to evaluate the immunogenic properties of the different recombinant proteins used. Plates (MaxiSorp^®^, Nunc, USA) were coated with 1 μg of protein per well in a final volume of 100 μl and incubated overnight at 4 °C. Afterward, the plates were blocked with 5% skim milk in PBST (PBSTM) and 100 μl of diluted (1:100) sera from pigs were then added to the plate and incubated for one hour at 37 °C. Plates were washed [3 times, 200 μl of PBS + 0.05% Tween 20 (PBST)] and 100 μl of goat anti-swine IgG (H&L) peroxidase conjugated antibody (1:5,000 dilution, Sigma-Aldrich, USA) was added to the plate and incubated for one hour at 37 °C. Wells were washed 3 times again, and the reaction was developed with 100 μl of 3,3′,5,5′-tetramethylbenzidine liquid substrate (TMB, Sigma-Aldrich) and left in the dark at room temperature for 10 minutes. The development was stopped with 50 μl of 2 M H_2_SO_4_ and the color reaction was analyzed at 450 nm in an ELISA reader (Synergy HI plate reader, BioTek^®^, USA). Experiments were performed in triplicate.

### Transferrin blocking assays

In order to evaluate whether antibodies from pigs immunized with either wild-type TbpB or a site directed mutant TbpB were able to interfere with wild-type TbpB ability to bind to pTf, a Tf blocking assay was developed. A volume of 1 ml of pig serum from each animal was passaged separately through a protein A Sepharose column (Sigma Aldrich, Canada), in order to bind serum IgG to the column and to elute out other serum components including pTf. Purified serum IgG samples were stored at 4 °C until use.

The Tf blocking assay was performed using the recombinant biotinylated TbpB of *H. parasuis* Nagasaki strain. 100 μl of crude extract obtained from *E. coli* (ER2566 strain) transformed with the expression vector containing the biotinylated TbpBs diluted 1:5 in PBST was added to the streptavidin ELISA plate (Greiner Bio-One, Austria) and incubated for 60 min at room temperature followed by blocking with 200 μl of PBSTM. Purified serum IgG antibodies were added to the wells at a dilution of approximately 1:3 of whole sera and incubated for 60 min at room temperature to allow the antibodies to bind. A 1:5,000 dilution of horseradish peroxidase (HRP)-pTf was added to the wells and incubated for 1 hour at room temperature. Plates were washed 3 times with 200 μl of PBST and developed as above. Successful blocking was considered as a decrease in reported OD of wells with sera compared to wells that received no sera in which pTf could bind without restriction.

### Statistical analysis

Differences amongst treatments were analyzed by the Kruskal-Wallis or one/two-way ANOVA followed by Tukey or Sidak post-test depending on the data normality assessed by Kolmogorov-Smirnov and Levene tests. The results are reported as means ± SEM and *P*-values < 0.05 were considered to be significant.

## Electronic supplementary material

Supplementary figures
