# Supplementary material for: New insights about functional and cross-reactive properties of antibodies generated against recombinant TbpBs of Haemophilus parasuis
Source: Sci Rep. 2017 Sep 4;7:10377. doi: 10.1038/s41598-017-10627-0 (PMC5583350; doi:10.1038/s41598-017-10627-0)
Supplement: Supplementary file 1 — Supplementary figures [file 41598_2017_10627_MOESM1_ESM.pdf]

New insights about functional and cross-reactive properties of antibodies generated against recombinant TbpBs of *Haemophilus parasuis*

Bibiana Martins Barasuol[a], João Antônio Guizzo[a], Jamie Elisabeth Fegan[b], Sonia Martínez-Martínez[c], Elías Fernando Rodríguez-Ferri[c], César Bernardo Gutiérrez-Martín[c], Luiz Carlos Kreutz[a], Anthony Bernard Schryvers[b], Rafael Frandoloso[a*]

[a] Laboratory of Microbiology and Advanced Immunology, Faculty of Agronomy and Veterinary Medicine, University of Passo Fundo, Passo Fundo 99052-900, Brazil.

[b] Department of Microbiology & Infectious Diseases, Faculty of Medicine, University of Calgary, Calgary, Alberta, Canada, T2N 4N1.

[c] Unidad de Microbiología e Inmunología, Departamento de Sanidad Animal. Facultad de Veterinaria, Universidad de León, 24007 - León, España

Supplementary Figure S1. Denaturing SDS-PAGE analysis of purified recombinant TbpB proteins. MW: molecular weight marker; 1: wild-type TbpB; 2: mutant TbpB (W176A); 3: mutant TbpB N lobe (TbpB-Nm); 4: TbpB C lobe (TbpB-C). The arrows indicate the estimated molecular weight of each protein derived from the amino acid sequence.

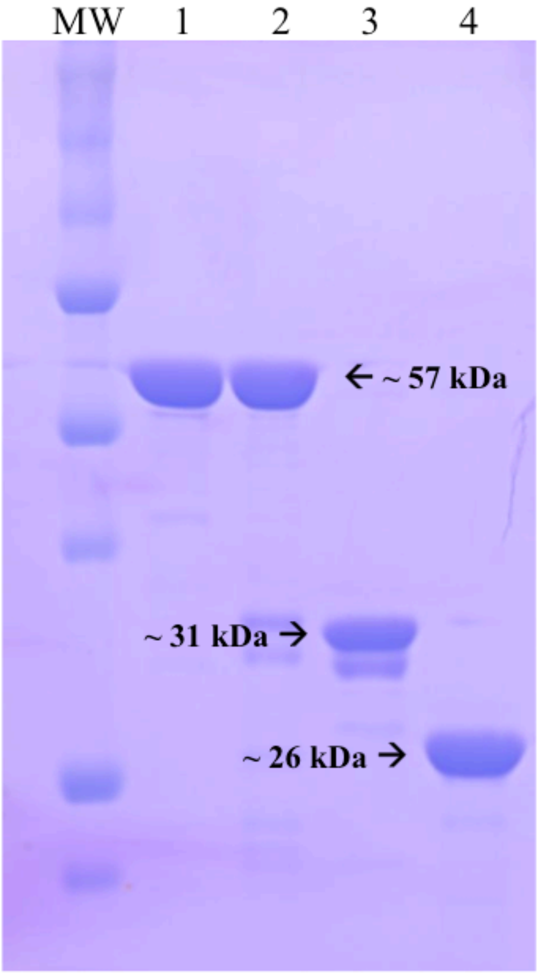

Supplementary Figure S2. Immunogenic analysis of TbpB-derived antigens. Pigs were immunized with recombinant wild-type TbpB, mutant TbpB (W176A), TbpB-Nm and TbpB-C. The level of antibody is indicated as the optical density (OD 450 nm). The data is expressed as the mean ± SEM of each group. The horizontal line represents the average OD from all piglets prior to immunization. Asterisks (*p<0.05, **p<0.01 and *** p<0.001) indicate statistical differences between the different time points within each group.

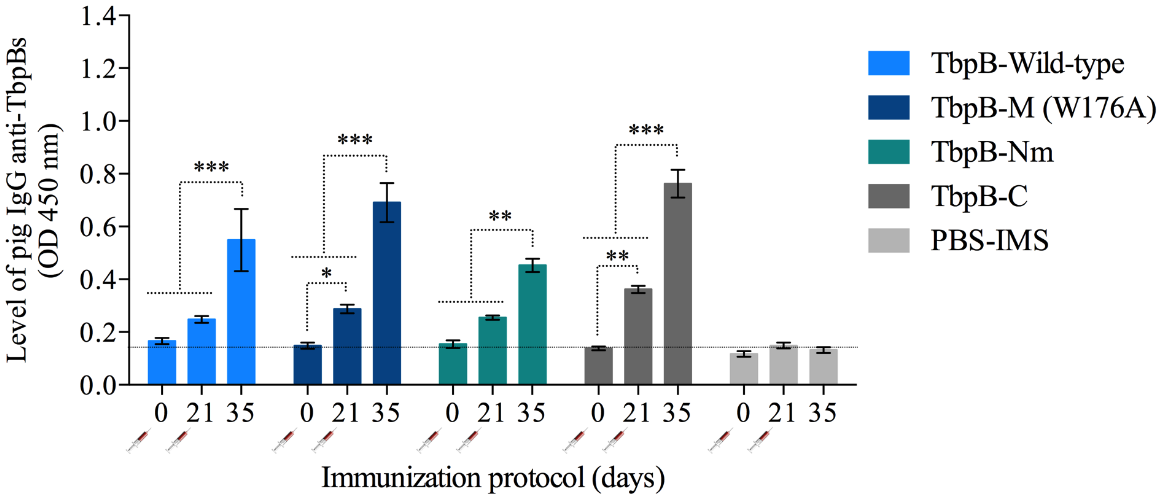

Supplementary Figure S3. Mouse TbpB-isotype specific antibody titres. Mice (n = 2/vaccine formulation) were immunized two times by intraperitoneal injection and blood samples were collected at fourteen days after the second immunization. Mouse immunoglobulin isotype (IgG1, IgG2a, IgG2b, IgG3, IgM and IgA) titres against wild-type TbpB were assessed by ELISA. The immunoglobulin titres are expressed as the reciprocal of the highest dilution that gave a positive OD reading. Each bar represents the mean ± SEM of each pair of immunized mice. Statistical differences were represented by asterisks (**p<0.01 and ***p<0.001).

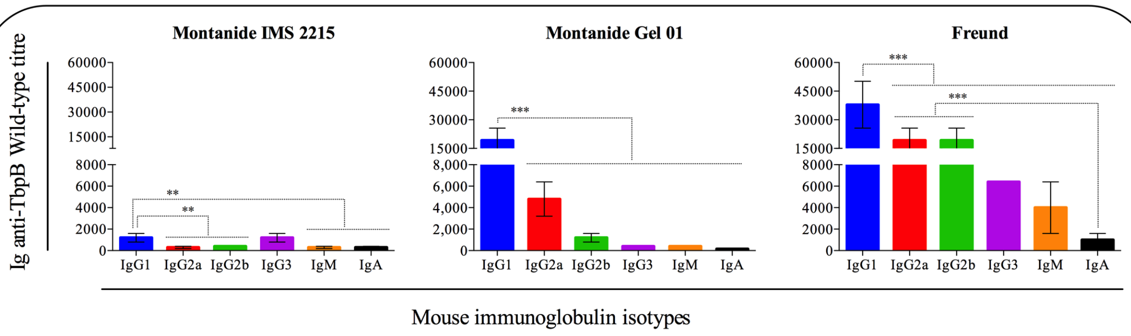

Mouse immunoglobulin isotype hierarchy related to serum bactericidal activity (SBA) proposed by Michaelsen, et al. [1]

IgG isotype	SBA
IgG1	+
IgG2a	++
IgG2b	+++
IgG3	++++

1 Michaelsen, T. E., Kolberg, J., Aase, A., Herstad, T. K. & Hoiby, E. A. The four mouse IgG isotypes differ extensively in bactericidal and opsonophagocytic activity when reacting with the P1.16 epitope on the outer membrane PorA protein of Neisseria meningitidis. *Scand J Immunol* **59**, 34-39 (2004).
